# Effects of the sport education model on table tennis skills, sport motivation, and social adaptation in high school students

**DOI:** 10.3389/fpsyg.2026.1756123

**Published:** 2026-02-02

**Authors:** Jianyu Tang, Xueyong Chen, Hongying Wang

**Affiliations:** College of Physical Education, Shanghai University of Sport, Shanghai, China

**Keywords:** high school students, social adaptation, sport education model, sport motivation, table tennis skills

## Abstract

**Objective:**

The Sport Education Model is widely regarded as a structured instructional framework capable of enhancing students’ motor skills, motivation to participate in physical activity, and socio-emotional competence. However, most existing studies focus on college students and major team sports. Empirical evidence for high school populations and individual racket sports such as table tennis remains limited.

**Methods:**

A quasi-experimental design was employed with two parallel classes from one high school. The experimental group received instruction based on the Sport Education Model, while the control group followed the traditional three-part lesson structure. Both groups experienced the same instructional duration and total class hours. After the intervention, students’ performance was assessed using table tennis skill tests, the Sport Motivation Scale, and the Social Adaptation Questionnaire. Independent-samples *t*-tests were used to compare post-intervention differences between groups.

**Results:**

In terms of sport skills, the experimental group showed significantly greater improvements in flat serve and backhand push-drive performance (*p* < 0.01), as well as significant gains in forehand attack and half-table two-point forehand drills (*p* < 0.05). In sport motivation, the experimental group demonstrated highly significant increases in intrinsic motivation for knowledge, accomplishment, and excitement (*p* < 0.01). Identified regulation and external regulation also improved significantly (*p* < 0.01), and introjected regulation reached a significant level (*p* < 0.05). Regarding social adaptation, the experimental group obtained significantly higher total scores than the control group (*p* < 0.01).

**Conclusion:**

Compared with the traditional teaching model, table tennis instruction based on the Sport Education Model substantially enhanced students’ sport-specific skills, learning motivation, self-efficacy, and social adaptation. These results indicate strong feasibility and practical value for implementing and promoting this instructional approach.

## 1 Introduction

The role of school physical education is shifting from single-skill instruction toward broader goals that foster students’ holistic development (Agans et al., 2024). These goals include cultivating enjoyment of physical activity, supporting personality and character development, and promoting social and psychological competencies (Dyson et al., 2024a; Jadwiszczak et al., 2025). As recent curriculum reforms highlight authenticity and active engagement in the learning process (Harvey et al., 2024), instructional models are increasingly expected to move beyond teacher-directed technical drills toward learning environments that provide sustained participation, authentic competition, and opportunities for students to take on multiple roles (Köycü et al., 2024; Bessa et al., 2023). However, in Chinese high schools, the traditional three-part instructional model remains dominant (Zhang et al., 2023). This model relies heavily on teacher demonstration, repetitive practice, and centralized control (Liu et al., 2024). Although effective for basic skill acquisition, it offers limited opportunities for students to experience authentic competition. Participation in competitive scenarios is often restricted to those with higher technical proficiency, leaving many students without meaningful game-like experiences and consequently limiting motivation and broader competence development (Sun et al., 2022).

Against this backdrop, researchers have increasingly turned their attention to alternative instructional models that can enhance engagement, competitive experience, and social learning. The Sport Education Model (SEM), a structured instructional framework widely adopted internationally, is built around a season-based unit that emphasizes stable teams, role diversification, and multiple formal competitions (Hastie, 1998). Within SEM, students act not only as players but also as referees, coaches, and scorekeepers, creating learning conditions that resemble authentic sport participation (Hastie et al., 2023). International evidence shows that SEM can significantly improve sport-specific skills, intrinsic motivation, responsibility, cooperation, and social adaptation across diverse educational settings (Harvey et al., 2024; Bessa et al., 2023).

Despite the growing global attention to SEM, two notable gaps remain. First, most existing studies focus on university students (Chen et al., 2020), while experimental research targeting high school students, who are at a critical developmental stage, is still limited (Shen et al., 2023). Second, the majority of SEM studies have concentrated on large-team sports such as basketball and volleyball (Liu et al., 2022). Research on individual or small-court sports, especially table tennis, is scarce (Hastie et al., 2023; Wallhead et al., 2022). Table tennis is well suited for season-based instruction due to its low space requirements, high accessibility, and rapid skill progression (Ortega-Zayas et al., 2025). Yet, under traditional teaching models, table tennis classes still rely heavily on demonstration and imitation, offering limited opportunities for cooperation, competition, and role-based engagement (Liu et al., 2024). Meanwhile, as table tennis continues to excel on the international stage and advances in global visibility, innovation in school-based instruction and talent development becomes increasingly important (Qian et al., 2022).

Introducing SEM into high school table tennis classes may create more authentic, engaging, and socially rich learning environments by integrating season structures, fixed teams, role responsibilities, and formal competitions. These features have the potential to enhance students’ skill performance, strengthen their motivation for physical activity, and promote the development of social adaptation.

This study aimed to comprehensively evaluate the effects of the Sport Education Model on high school students’ table tennis skills, sport motivation, and social adaptation, by implementing a structured SEM intervention and comparing it with the traditional three-part lesson format. The research specifically examined whether SEM could enhance technical skill proficiency, intrinsic and extrinsic motivation, and socio-emotional competence in a small-court sport context. Therefore, the present study aims to investigate the impact of SEM-based table tennis instruction on students’ skill development, motivation, and social adaptation, providing empirical evidence for its feasibility and effectiveness in Chinese high schools.

## 2 Materials and methods

### 2.1 Study design and procedure

This study followed the principles of the Declaration of Helsinki and was approved by the Ethics Committee of Shanghai University of Sport. All participants provided informed consent. A quasi-experimental design with intact classes was used to compare the effects of the Sport Education Model and the traditional three-part instructional model in high school table tennis lessons. Both groups received 20 sessions of table tennis instruction, each lasting 40 min. The research followed a pretest–intervention–posttest framework consisting of baseline assessments, differentiated instructional interventions, post-intervention testing, and data analysis.

The experimental group followed a complete SEM season structure, including a preparation phase, a skill development phase, a formal competition phase, and a culminating event. In the preparation phase, teachers introduced SEM concepts and class expectations, formed heterogeneous fixed teams, assigned roles such as captain, referee, and statistician, and organized team identity design. During the skill development phase, instruction combined teacher guidance with peer-assisted practice on key techniques, including flat serve, forehand attack, backhand push-drive, and half-table two-point drills. Students were encouraged to organize practice autonomously and develop contextual understanding through rule learning and officiating practice. The formal competition phase consisted of preseason matches, regular-season rounds, and playoffs, combining round-robin and elimination formats. Students took responsibility for officiating, recording results, and engaging in tactical planning, while teachers provided oversight and classroom management. The culminating phase included championship matches, skill challenges, award ceremonies, and team reflection.

The control group followed the conventional three-part lesson structure commonly used in Chinese high schools, consisting of warm-up, basic practice, and closure. Teachers led all instructional tasks through demonstrations, group drills, and direct feedback. The sequence of technical instruction matched that of the experimental group, but student roles and competitive activities were substantially limited.

### 2.2 Participants

A total of 116 first-year high school students (59 males and 57 females; mean age 15.3 ± 0.4 years) were recruited from table tennis elective classes at a single high school in Shanghai using convenience sampling. Two intact classes were selected and assigned to the experimental group (*n* = 58) or control group (*n* = 58) based on existing class allocations, forming a quasi-experimental, non-randomized parallel group design. This design allowed for comparison of the Sport Education Model and traditional instructional approaches while maintaining natural classroom structures. Intact classes were used due to school logistical constraints and to minimize disruption to existing class schedules. None had prior specialized table tennis training, and all were physically able to participate. No significant baseline differences were found between groups in table tennis skills, sport motivation, or social adaptation (*p* > 0.05). Written informed consent was obtained from all students and their guardians.

### 2.3 Assessment of table tennis skills, sport motivation, and social adaptation

#### 2.3.1 Table tennis skill assessment

Table tennis skills were assessed using Level 3 standards from the Youth Sport Skill Level Standards and Test Methods (Table Tennis) (Shanghai University of Sport, 2017). The assessment included four subtests (25 points each, total 100 points). Higher scores indicated better skill proficiency.

1. Flat serve: 20 serves, scoring based on successful hits to a designated target area.
2. Backhand push-drive: 40 strokes, scoring based on valid target hits.
3. Forehand attack: 40 consecutive strokes, recorded by target hits.
4. Half-table two-point forehand drill: 40 strokes, evaluated similarly.

All tests were conducted by trained instructors following standardized procedures to ensure scoring consistency.

#### 2.3.2 Sport motivation assessment

Sport motivation was measured using the Chinese version of the Sport Motivation Scale (SMS) (Goudas et al., 1994). The scale includes intrinsic motivation (knowledge, accomplishment, excitement), extrinsic motivation (identified regulation, introjected regulation, external regulation), and amotivation. Students rated their motivational states related to sport participation, interest, skill learning, and value recognition.

#### 2.3.3 Social adaptation assessment

Social adaptation was measured using the Social Adaptation Diagnostic Scale (Zheng, 1999), which evaluates domains such as emotional regulation, interpersonal interaction, behavioral control, and social participation. Students rated each item based on their actual experiences. The scale assessed baseline social functioning and post-intervention development.

### 2.4 Statistical analysis

**Figure 1:**
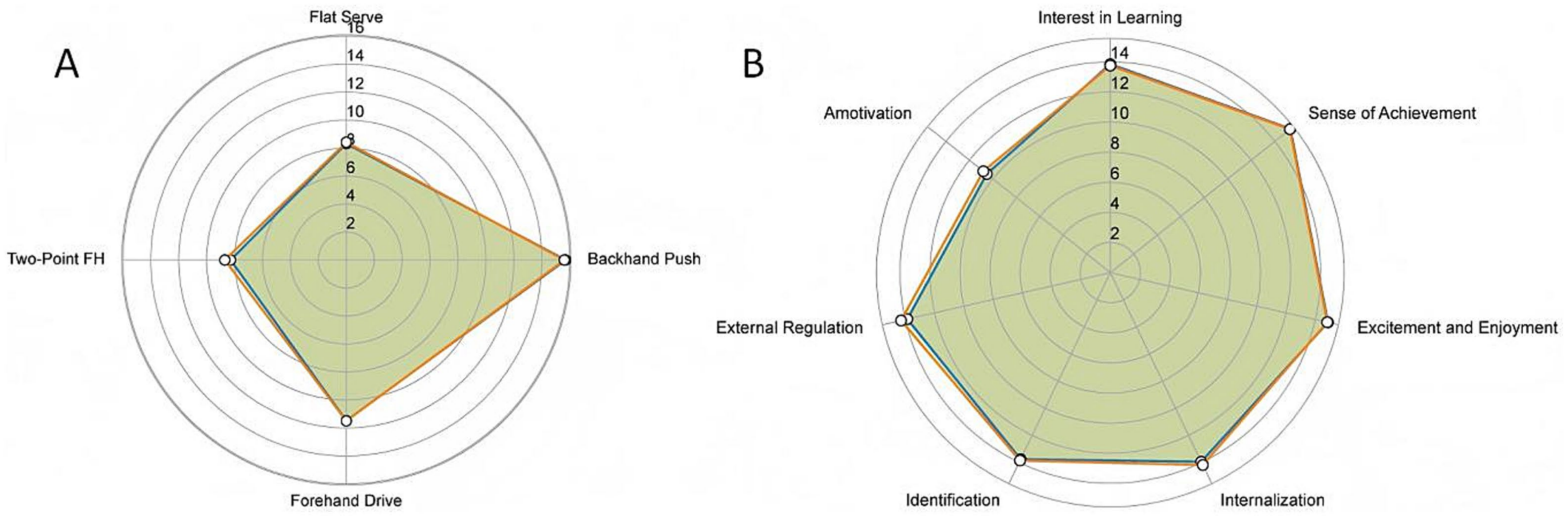
Baseline performance of table tennis skills and motivational dimensions between the experimental and control groups. Panel **(A)** shows the four table tennis skill indicators. Panel **(B)** presents the seven motivational dimensions.

All analyses were conducted using SPSS 26.0. Normality was tested using the Shapiro–Wilk test. Paired-samples *t*-tests were used to evaluate within-group changes, and independent-samples *t*-tests were used to compare groups. The significance level was set at *α* = 0.05. Cohen’s d was calculated to assess effect sizes (see Figure 1).

## 3 Results

### 3.1 Baseline comparisons

Before the intervention, no significant differences were found between groups in sex distribution, age, table tennis skill performance, sport motivation dimensions, or social adaptation scores (all *p* > 0.05). The groups demonstrated comparable levels across all four table tennis skill tests, intrinsic and extrinsic motivation, amotivation, and social adaptation. These findings indicate strong baseline equivalence between groups (Table 1).

**Table 1:** Baseline characteristics of the experimental and control groups.

Characteristics		Experimental group (*M* ± SD)	Control group (*M* ± SD)	Test statistics	*p*
Flat serve		8.32 ± 3.29	8.42 ± 3.06	−0.179	0.859
Backhand push		15.71 ± 5.14	15.61 ± 4.33	0.119	0.905
Forehand drive		11.48 ± 3.05	11.50 ± 3.34	−0.030	0.976
Half-table forehand two-point drill		8.29 ± 2.72	8.70 ± 2.75	−0.795	0.428
Intrinsic motivation	Interest in learning	13.86 ± 3.64	13.76 ± 3.55	0.086	0.932
	Achievement	15.33 ± 3.34	15.29 ± 3.04	0.048	0.962
	Excitement and enjoyment	14.86 ± 2.67	14.81 ± 3.11	0.053	0.958
Extrinsic motivation	Internalization	13.95 ± 3.83	14.19 ± 3.44	−0.212	0.833
	Identification	13.76 ± 2.95	13.86 ± 2.56	−0.112	0.911
	External regulation	13.86 ± 3.50	14.29 ± 3.51	−0.396	0.694
Amotivation		10.52 ± 2.60	10.81 ± 3.04	−0.327	0.745
Total social adaptation score		11.08 ± 12.31	11.54 ± 12.07	−0.137	0.892

Values are presented as mean ± standard deviation (SD). Independent samples t tests were used to compare the baseline scores between groups.

### 3.2 Within-group changes

**Figure 2:**
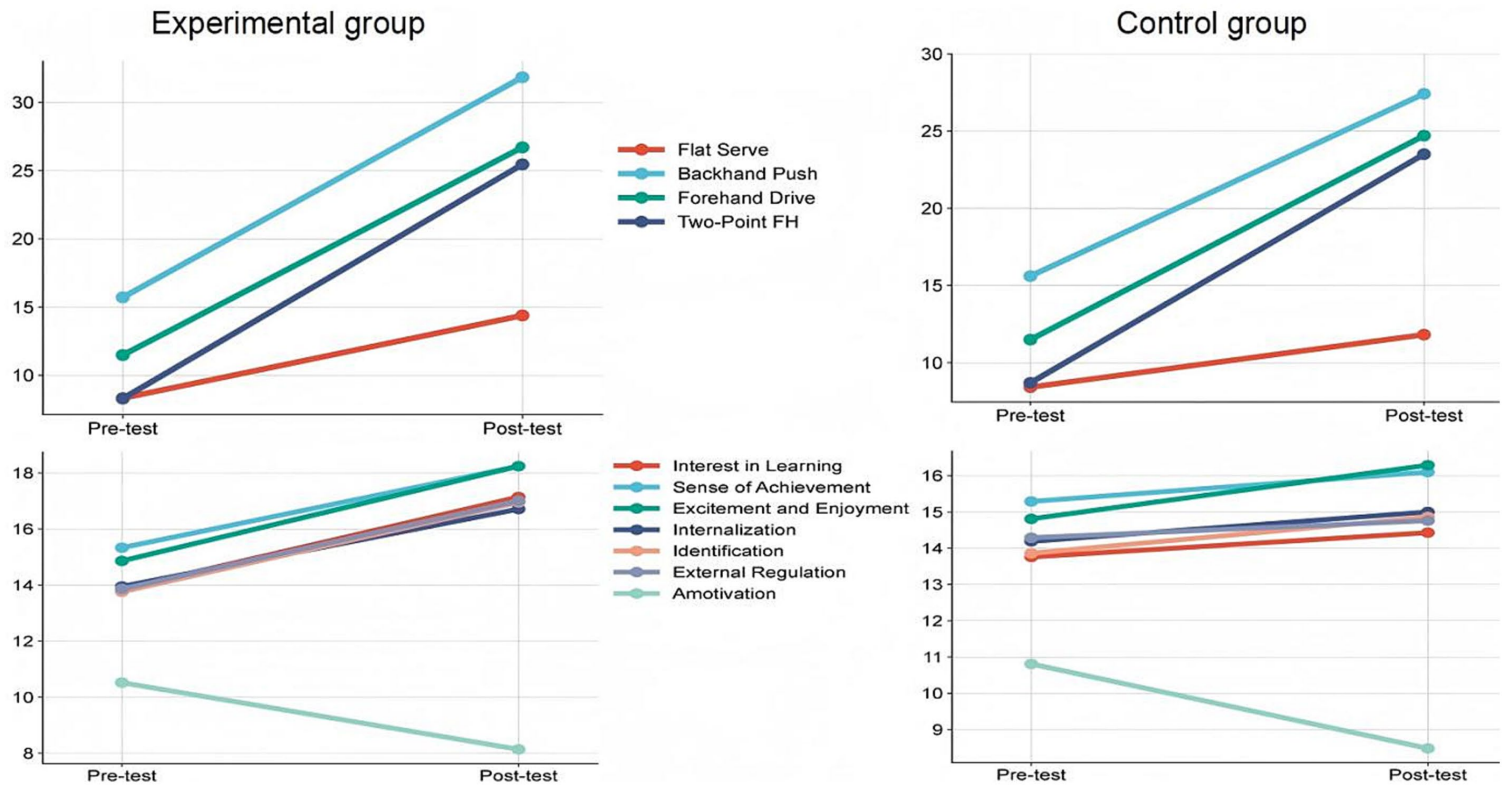
Pre- and post-test changes in table tennis skills and motivation in the experimental and control groups.

After the 20-lesson intervention, both groups showed improvements in sport-specific skills, sport motivation, and social adaptation compared with baseline. However, the gains were substantially larger in the experimental group. Significant increases were observed in the experimental group for flat serve, backhand push, forehand drive, and the half-table two-point forehand drill (*p* < 0.001), with effect sizes ranging from medium to large. Detailed results are presented in Table 2 and visualized in Figure 2.

**Table 2:** Pre- and post-test performance of table tennis skills, motivation, and social adaptation in the experimental group.

Characteristics		Pre-test	Post-test	Mean difference	*p*
Flat serve		8.32 ± 3.29	14.38 ± 2.63	6.06	<0.001***
Backhand push		15.71 ± 5.14	31.84 ± 3.95	16.13	<0.001***
Forehand drive		11.48 ± 3.05	26.70 ± 5.48	15.22	<0.001***
Half-table forehand two-point drill		8.29 ± 2.72	25.45 ± 4.01	17.16	<0.001***
Intrinsic motivation	Interest in Learning	13.86 ± 3.64	17.14 ± 2.67	3.28	<0.001***
	Achievement	15.33 ± 3.34	18.24 ± 1.48	2.91	<0.001***
	Excitement and Enjoyment	14.86 ± 2.67	18.24 ± 1.26	3.38	<0.001***
Extrinsic motivation	Internalization	13.95 ± 3.83	16.71 ± 2.31	2.76	<0.001***
	Identification	13.76 ± 2.95	16.95 ± 1.99	3.19	<0.001***
	External regulation	13.86 ± 3.50	17.00 ± 2.32	3.14	<0.001***
Amotivation		10.52 ± 2.60	8.14 ± 2.56	−2.38	<0.001***
Total social adaptation score		11.08 ± 12.31	29.15 ± 5.43	18.07	<0.001***

Values are presented as mean ± SD. Paired samples *t* tests were used to examine pre–post differences within the experimental group. Positive mean differences indicate improvement.

*p < 0.05, **p < 0.01, ***p < 0.001 indicate statistical significance.

For sport motivation, the experimental group demonstrated significant improvements in intrinsic motivation, including knowledge, accomplishment, and excitement (*p* < 0.01), as well as in identified and introjected regulation within extrinsic motivation. Amotivation decreased significantly, indicating that students developed more autonomous forms of motivation during the learning process. Social adaptation also improved significantly across emotional regulation, interpersonal interaction, and cooperative communication (*p* < 0.01), as summarized in Table 2.

The control group showed modest improvements between pretest and posttest, particularly in skill performance and several motivational dimensions, though most effect sizes were small. These patterns are reported in Table 3 and displayed in Figure 2 for direct comparison.

**Table 3:** Pre- and post-test performance of table tennis skills, motivation, and social adaptation in the control group.

Characteristics		Pre-test	Post-test	Mean difference	*p*
Flat serve		8.42 ± 3.06	11.82 ± 3.04	3.40	<0.001***
Backhand push		15.61 ± 4.33	27.41 ± 4.86	11.80	<0.001***
Forehand drive		11.50 ± 3.34	24.70 ± 5.33	13.20	<0.001***
Half-table forehand two-point drill		8.70 ± 2.75	23.50 ± 4.12	14.80	<0.001***
Intrinsic motivation	Interest in learning	13.76 ± 3.55	14.43 ± 2.80	0.67	0.064
	Achievement	15.29 ± 3.04	16.10 ± 1.97	0.81	0.040*
	Excitement and enjoyment	14.81 ± 3.11	16.29 ± 2.05	1.48	0.001**
Extrinsic motivation	Internalization	14.19 ± 3.44	15.00 ± 2.55	0.81	0.111
	Identification	13.86 ± 2.56	14.86 ± 2.10	1.00	0.020**
	External regulation	14.29 ± 3.51	14.76 ± 2.59	0.47	0.204
Amotivation		10.81 ± 3.04	8.48 ± 2.34	−2.33	<0.001***
Total social adaptation score		11.54 ± 12.07	20.62 ± 8.23	9.08	<0.001***

Values are presented as mean ± SD. Paired samples *t* tests were used to examine pre–post differences within the control group. Positive mean differences indicate improvement.

*p < 0.05, **p < 0.01, ***p < 0.001 indicate statistical significance.

### 3.3 Between-group differences after the intervention

Posttest comparisons revealed that the experimental group performed significantly better than the control group on all four sport-specific skill indicators (*p* < 0.01). In terms of sport motivation, the experimental group scored significantly higher on all three intrinsic motivation dimensions and selected extrinsic motivation dimensions. No significant difference was observed between groups for amotivation (*p* = 0.661).

**Figure 3:**
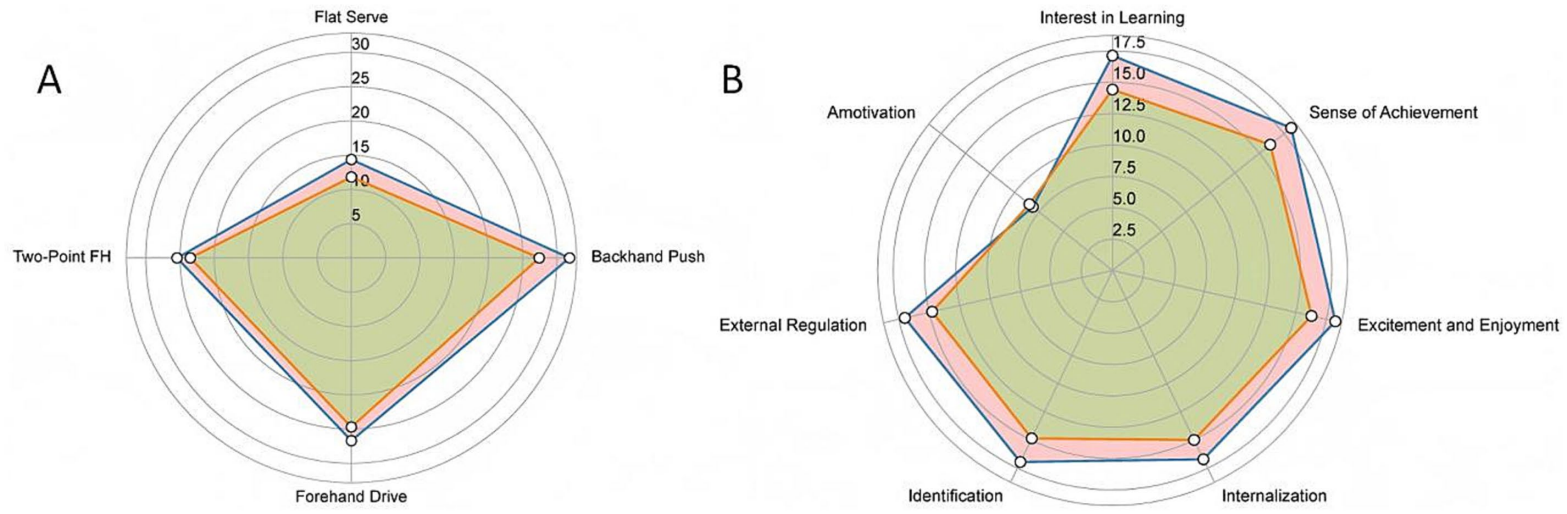
Post-test differences in table tennis skills and motivation between experimental and control groups. Panel **(A)** shows the four table tennis skill indicators. Panel **(B)** presents the seven motivational dimensions.

For social adaptation, the experimental group showed significantly higher total scores than the control group, indicating that the SEM-based instruction produced stronger gains in emotional regulation, cooperative communication, and interpersonal functioning. Overall, the experimental group exhibited larger posttest improvements in skills, motivation, and social adaptation. Corresponding effect sizes indicate medium-to-large effects under the SEM intervention, whereas improvements in the traditional instruction condition were generally small (Table 4; Figure 3).

**Table 4:** Post-test comparison of table tennis skills, motivation, and social adaptation between the experimental and control groups.

Characteristics		Experimental group	Control group	Mean difference	Test statistics	*p*
Flat serve		14.38 ± 2.63	11.82 ± 3.04	2.56	4.752	<0.001***
Backhand push		31.84 ± 3.95	27.41 ± 4.86	4.43	5.292	<0.001***
Forehand drive		26.70 ± 5.48	24.70 ± 5.33	2.00	2.957	0.033*
Half-table forehand two-point drill		25.45 ± 4.01	23.50 ± 4.12	1.95	2.532	0.013*
Intrinsic motivation	Interest in learning	17.14 ± 2.67	14.43 ± 2.80	2.71	3.213	0.003**
	Achievement	18.24 ± 1.48	16.10 ± 1.97	2.14	3.982	<0.001***
	Excitement and enjoyment	18.24 ± 1.26	16.29 ± 2.05	1.95	3.713	0.001**
Extrinsic motivation	Internalization	16.71 ± 2.31	15.00 ± 2.55	1.71	2.286	0.028*
	Identification	16.95 ± 1.99	14.86 ± 2.10	2.09	3.318	0.002**
	External regulation	17.00 ± 2.32	14.76 ± 2.59	2.24	2.95	0.005**
Amotivation		8.14 ± 2.56	8.48 ± 2.34	−0.34	−0.441	0.661
Total social adaptation score		29.15 ± 5.43	20.62 ± 8.23	8.53	4.415	<0.001

Values are presented as mean ± SD. Independent samples t tests were used to compare post-test outcomes between the experimental and control groups.

*p < 0.05, **p < 0.01, ***p < 0.001 indicate statistical significance.

### 3.4 Integrated presentation of intervention effects

**Figure 4:**
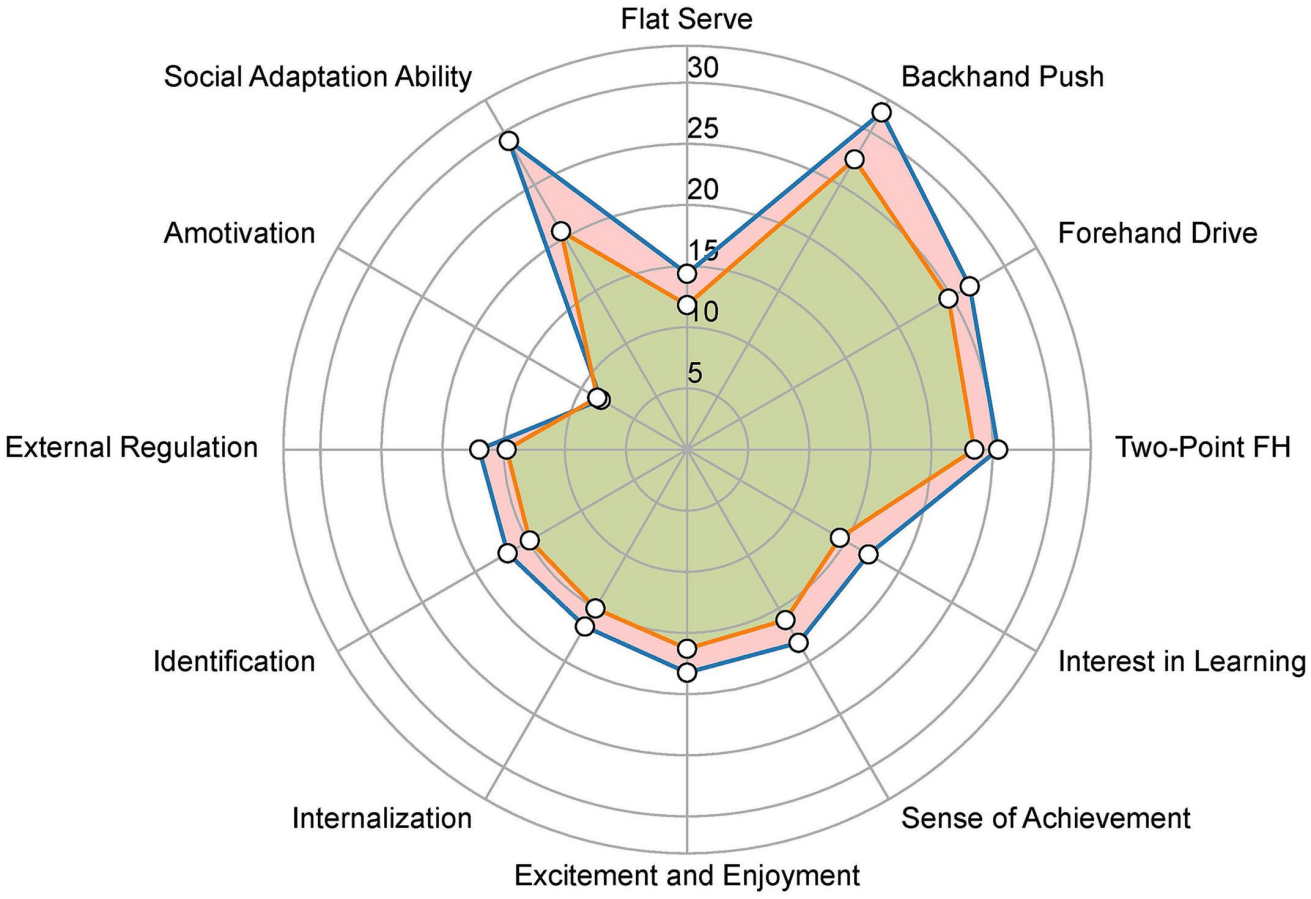
Integrated radar plot of skills, motivation, and social adaptation in experimental vs. control groups.

To provide a multidimensional view of the intervention effects, skill performance, motivation, and social adaptation were integrated into a radar plot. Each axis of Figure 4 represents one outcome domain, with values corresponding to post-test scores for the experimental and control groups. The experimental group outperformed the control group across all three domains, producing a stable pattern of multi-domain enhancement (Figure 4). These results suggest that the structured nature of SEM, including role responsibility and team-based cooperation, generates synergistic benefits across technical, motivational, and psychosocial outcomes.

## 4 Discussion

The findings of this study showed that the experimental group exhibited significant advantages in sport-specific skill performance, particularly in flat serve and backhand push (*p* < 0.01), and also demonstrated notable improvements in forehand drive and the half-table two-point forehand drill (*p* < 0.05). In terms of sport motivation, the experimental group showed significant increases in the three dimensions of intrinsic motivation, including knowledge, accomplishment, and excitement (*p* < 0.01). Identified regulation and external regulation also increased significantly (*p* < 0.01), and introjected regulation reached statistical significance (*p* < 0.05). Social adaptation scores were substantially higher among students in the experimental group compared with those in the control group (*p* < 0.01). These results align with international research trends and provide new empirical evidence for the application of the Sport Education Model (SEM) in small-court sports, particularly table tennis, within Chinese high school contexts. Overall, the experimental group exhibited larger posttest improvements in skills, motivation, and social adaptation. Corresponding effect sizes (Cohen’s d) for the SEM intervention typically ranged from 0.60 to 1.25 across outcome domains, indicating medium to large effects and demonstrating a strong practical impact of the intervention, whereas improvements in the traditional instruction condition were generally small.

Regarding skill development, the present findings indicate that SEM’s season-based structure, stable team organization, and formal competition cycles markedly enhanced students’ ability to transfer skills and perform in context-rich game situations. This pattern is consistent with recent randomized controlled trials and systematic reviews in ball sports that highlight SEM’s capacity to improve decision making, contextualized technique execution, and performance stability (Zhang et al., 2024; Farias et al., 2019). Prior studies have shown that SEM enhances performance not merely through repetitive technical drills but through purposeful, opposition-based practice that strengthens the perception–decision–action cycle (Zhang et al., 2024; Calle et al., 2024; Tokay and Akil, 2025). The current study extends these findings to a fast-paced racket sport, demonstrating that SEM supports students’ real-game competence even in environments requiring rapid perceptual and tactical responses. Compared with the long-standing traditional approach to table tennis instruction in China, which emphasizes imitation-based drills and isolated technique practice (Zhang et al., 2023), this study provides evidence that SEM offers a viable pathway for addressing the prevalent pedagogical challenge whereby students “execute techniques well but struggle in actual matches.”

In terms of sport motivation, SEM exerted particularly robust effects. Unlike teacher-centered, demonstration–practice instructional formats, SEM fosters high autonomy, high engagement, and high challenge through stable teams, role responsibilities, and authentic competition. These features directly stimulate students’ perceived competence, autonomy, and relatedness, which are core psychological needs according to self-determination theory (Moreno-Murcia et al., 2024; Sun and Li, 2024). The structural design of SEM, incorporating role rotation, peer feedback, and sustained competitive environments, effectively supports these needs (Harvey et al., 2024; Dai et al., 2024). Recent evidence further suggests that motivational gains produced by SEM may transfer across contexts, contributing to increased physical activity participation beyond the classroom (Choi et al., 2024). In this study, significant gains in intrinsic motivation as well as in more autonomous forms of extrinsic motivation (identified and introjected regulation) suggest that high school students not only experienced more positive emotional engagement but also developed deeper value internalization related to table tennis. Such motivational restructuring has important implications for long-term sport participation and behavioral sustainability.

With respect to social adaptation, the results demonstrate that SEM functions as an effective platform for social and emotional learning (Neslihan Arikan, 2020; Jadwiszczak et al., 2025; Dyson et al., 2024b; Manninen and Campbell, 2022). Unlike traditional teacher-centered lessons with limited peer interaction, SEM’s stable team structure, multi-role task system, and competitive activities create continuous opportunities for communication, negotiation, conflict management, and collaborative decision making (Bessa et al., 2023; Jadwiszczak et al., 2025). Prior research highlights that SEM elements such as team responsibility systems and process-oriented assessment contribute to improved rule awareness, social initiative, and collective goal orientation (Kao, 2019; Song and Xue, 2022). The present findings show that even in a small-court sport like table tennis, which is often assumed to offer limited social learning opportunities, SEM can significantly enhance students’ emotional regulation, interpersonal communication, and cooperative functioning through repeated role engagement and team-based collaboration. These findings expand the known applicability of SEM across different sport types and challenge the conventional assumption that table tennis has limited potential for fostering social development.

The theoretical contribution of this study lies in contextualizing adolescents’ motivational development and social adaptation within a structured pedagogical framework. First, the findings confirm that SEM’s team-based organization, role responsibilities, and authentic performance assessments create a highly autonomy-supportive learning environment that fulfills students’ needs for competence, autonomy, and relatedness. This environment fosters intrinsic motivation and internalization processes, providing cross-context evidence for the applicability of self-determination theory in small-court, technique-intensive physical education settings. Second, interpreting SEM through the lens of social and emotional learning reveals that stable peer groups and repeated cooperative interactions offer adolescents authentic social environments to practice emotional regulation, collaboration, and social responsibility. Physical education can therefore be conceptualized as a “micro-social ecosystem” in which structured role participation and collective task engagement promote the continuous development of motivation and psychosocial competencies. By integrating structured sport pedagogy with motivational and developmental psychology, this study contributes a more comprehensive theoretical explanation of SEM’s mechanisms and clarifies how it supports multidimensional student development in real-world classroom contexts.

Despite the strengths of this study, several limitations should be acknowledged. First, the relatively short duration of the intervention limits the ability to evaluate long-term behavioral and psychological outcomes. Second, the analysis did not stratify results by gender, baseline skill level, or initial motivational profile, factors that may influence differential responsiveness to SEM. Future research should adopt multi-site designs, expand sample sizes, and incorporate mixed methods such as classroom observations, behavioral tracking, and qualitative interviews to more fully capture SEM’s fidelity, underlying mechanisms, and sustained effects.

## 5 Conclusion

Compared with traditional instruction, the Sport Education Model significantly enhanced students’ sport-specific skills, learning motivation, self-efficacy, and social adaptation. These findings indicate that SEM is a feasible and effective pedagogical model with strong potential for broader implementation. Table tennis instruction represents not only a technical learning process but also an avenue for strengthening student interest, building confidence, and fostering social interaction. Future physical education programs should continue to refine structured instructional design and expand opportunities for interaction, cooperation, and feedback to maximize the educational value of PE in cultivating multidimensional student development.

## Data Availability

The raw data supporting the conclusions of this article will be made available by the authors, without undue reservation.
